# Reply to Rayman et al. Comment on “Ambra et al. Could Selenium Supplementation Prevent COVID-19? A Comprehensive Review of Available Studies. *Molecules* 2023, *28*, 4130”

**DOI:** 10.3390/molecules29112468

**Published:** 2024-05-24

**Authors:** Roberto Ambra, Sahara Melloni, Eugenia Venneria

**Affiliations:** Consiglio per la Ricerca in Agricoltura e l’Analisi dell’Economia Agraria (CREA)—Research Centre for Food and Nutrition, Via Ardeatina 546, 00178 Rome, Italy; sahara.melloni@crea.gov.it (S.M.); eugenia.venneria@crea.gov.it (E.V.)

We thank the Authors of the Comment (AC) [1] for their feedback. However, we have found some misleading observations, starting on selenium’s role and effects: more than 50 selenoprotein families are known [2], and selenium is essential for the activity of selenoenzymes [3]. Thus, even if we agree that the sentence “selenium modulates the biochemical activity of several enzymes” could be a simplifying opinion, we believe it is not a “misrepresentation” of the role of selenium. Moreover, our scope was not to describe the effects of selenium on human mortality. Our aim was to give an evaluation of the published works on selenium and COVID-19.

With respect to the title, the different types of articles included in our review are a clear demonstration that we did not refer *exclusively* to the possible effects of selenium on COVID-19 *infection*. Moreover, as stated in the Methodology section, the collection of articles was performed in December 2022. This is why the mentioned work from Larvie et al., published in February 2023, is missing in our review. Regarding Demircan’s paper, we kindly bring to AC’s attention that this article is a review and that it includes scientific articles that we considered in our review.

The AC accuse us of having defined one of their studies as meaningless; such a statement has never been declared. As stated by AC, they “acknowledged that our study has an important number of limitations”. We have simply given a critical reading of the works without offending. As ours is a critical review, we did not focus on the main findings of each report, and we just highlighted the critical aspects. Thus, introducing “their main finding (the association of cure rate and background hair selenium)”, and furthermore lacking many of the confounding factors (as AC themselves declare in their publication) that could affect the results, was outside of our scope. However, similarly to the AC, we agree that further research is needed before we can establish that a supplementation of selenium is fundamental against COVID-19. This was indeed the topic of our review, and we did not mean to question the importance of the role of selenium in human health.

We thank the AC for clarifying that the two hair selenium reports about the Hubei province, in Table 2 of their reference 10, are exclusively from inhabitants of the city of Wuhan. Instead, we find the argument brought about the correlation for cities outside Hubei to be confounding. In fact, the AC themselves wrote in their article that “the outcome data for Hubei and outside-Hubei are statistically distinct, necessitating the separate treatment of Hubei (where mortality was much higher) and outside-Hubei in our subsequent analyses”. Our point is simpler: in our opinion, AC contradicts in the text their own data of Table S1, i.e., Enshi is NOT an exception to the high mortality rate of Hubei Province. Moreover, the data on Xianning appear to be more reliable due to more medical cases (868 vs. 250 patients).

With respect to the “remarkable results reported by Melinda Beck”, as we wrote in the review, our critical point is that no indication is given, in the publication in question, as to how much selenium was administered to animals with the diet.

Regarding Du Laing et al., we thank AC for noticing our error in the year of ethics approval and we apologize for the typo. Regarding data availability upon request, we believe that the purpose of a review is to analyze published data. In any case, the availability of COVID-19 vaccination information it is not mentioned anywhere in the article nor in the Data Availability Statement. With respect to selenium thresholds, we were misled by that of 55.2 µg/L, used arbitrary in the article as a “deficiency threshold” below which “Not a single patient with a sufficient Se status succumbed to COVID-19 in the senior patient group above 65 years of age”, which appeared to us to be contradictory from what was depicted in Figure 2, i.e., at least two patients having had, at admission, a selenium concentration over this amount and dying at the end of the study. With respect to statistics, we specifically pointed to the lack of linearity between selenium and SELENOP status and disease severity. In fact, based on the data presented, there is no statistical significancy both between the classifications Mild–Mod vs. Severe and between Critical vs. Death. Moreover, we have also pointed to the authors’ claim of an increased death risk for selenium deficiency in patients with comorbidities, which, in our opinion, appeared unsupported in the table dealing with mortality (Table 3) because of the p-values presented. Finally, the detail on the reduced number of subjects used to calculate the mortality risk was not a criticism, it was just an underlining for the readers of the review, given that a larger number is reported a few lines above.
